# Comparison of the Effect of Different Conditioning Media on the Angiogenic Potential of Hypoxia Preconditioned Blood-Derived Secretomes: Towards Engineering Next-Generation Autologous Growth Factor Cocktails

**DOI:** 10.3390/ijms24065485

**Published:** 2023-03-13

**Authors:** Philipp Moog, Jessica Hughes, Jun Jiang, Lynn Röper, Ulf Dornseifer, Arndt F. Schilling, Hans-Günther Machens, Ektoras Hadjipanayi

**Affiliations:** 1 Experimental Plastic Surgery, Clinic for Plastic, Reconstructive and Hand Surgery, Klinikum Rechts der Isar, Technische Universität München, D-81675 Munich, Germany; 2 Department of Plastic, Reconstructive and Aesthetic Surgery, Isar Klinikum, D-80331 Munich, Germany; 3 Department of Trauma Surgery, Orthopedics and Plastic Surgery, Universitätsmedizin Göttingen, D-37075 Göttingen, Germany; 4 Department of Plastic, Reconstructive and Aesthetic Surgery, Lefkos Stavros Hospital, 115 28 Athens, Greece

**Keywords:** peripheral blood cells, blood-derived therapy, hypoxia, angiogenesis, hypoxia preconditioned plasma, hypoxia preconditioned serum, hypoxia preconditioned media, nutrition, lactate

## Abstract

Hypoxia Preconditioned Plasma (HPP) and Serum (HPS) are regenerative blood-derived growth factor compositions that have been extensively examined for their angiogenic and lymphangiogenic activity towards wound healing and tissue repair. Optimization of these secretomes’ growth factor profile, through adjustments of the conditioning parameters, is a key step towards clinical application. In this study, the autologous liquid components (plasma/serum) of HPP and HPS were replaced with various conditioning media (NaCl, PBS, Glucose 5%, AIM V medium) and were analyzed in terms of key pro- (VEGF-A, EGF) and anti-angiogenic (TSP-1, PF-4) protein factors, as well as their ability to promote microvessel formation in vitro. We found that media substitution resulted in changes in the concentration of the aforementioned growth factors, and also influenced their ability to induce angiogenesis. While NaCl and PBS led to a lower concentration of all growth factors examined, and consequently an inferior tube formation response, replacement with Glucose 5% resulted in increased growth factor concentrations in anticoagulated blood-derived secretomes, likely due to stimulation of platelet factor release. Medium substitution with Glucose 5% and specialized peripheral blood cell-culture AIM V medium generated comparable tube formation to HPP and HPS controls. Altogether, our data suggest that medium replacement of plasma and serum may significantly influence the growth factor profile of hypoxia-preconditioned blood-derived secretomes and, therefore, their potential application as tools for promoting therapeutic angiogenesis.

## 1. Introduction

Complete restoration of physiological tissue architecture is the ultimate goal of regenerative medicine research [1]. Taking into account that wounds naturally heal via a set of complex and interactive processes, including hemostasis, inflammation, proliferation, and remodeling [2,3], gives an idea of the intricate nature of tissue repair. Research on regenerative therapies commonly focuses on stimulating angiogenesis (formation of new blood vessels) and improving tissue perfusion in order to provide an adequate supply of oxygen/nutrients to the wound bed, which presents an absolute prerequisite for optimal cellular function. Employing the body’s own resources towards this goal, via, for example, the utilization of autologous blood-derived products, is becoming a more favorable approach, since it overcomes the limitations imposed by our incomplete understanding of these complex mechanisms, while also harnessing the physiological response that is necessary for avoiding unwanted side effects [4]. 

In our previous work, we showed that angiogenesis and lymphangiogenesis have symbiotic roles in wound healing, since they both appear to rely on overlapping growth factor mechanisms [5,6,7,8]. There is now strong evidence indicating that hypoxia preconditioned blood-derived secretomes could constitute a new generation of autologous, bioactive compositions that can supply the necessary biochemical signals for stimulating angiogenesis/lymphangiogenesis, thus driving wound healing to completion [5,6,8,9,10,11,12,13,14]. These growth factor compositions can be obtained through the method of hypoxia-adjusted in vitro preconditioning of peripheral blood cells (PBCs), first proposed by Hadjipanayi and Schilling [5,10,11,12]. Conditioning PBCs under the very same conditions encountered within a wound microenvironment, i.e., physiological temperature and hypoxia, offers a means for optimizing the angiogenic potential of Hypoxia Preconditioned Plasma (HPP) and Hypoxia Preconditioned Serum (HPS), which can be differentially prepared by adjusting blood coagulation prior to hypoxic conditioning [5,6,9,10,11,12,15]. More specifically, we have shown that the angiogenic potential of blood-derived secretomes is defined by the complex stoichiometry of their component pro- and anti-angiogenic factor proteins, rather than the concentration of one or more individual growth factors [6,8,12,15]. The angiogenic potency of hypoxia preconditioned secretomes is further highlighted by the fact that they maintain their pro-angiogenic activity in vitro, even when they are prepared from peripheral blood that has been obtained from patients who receive oral anticoagulation due to underlying vascular pathology or who suffer from diabetes mellitus [16].

The ability to control the growth factor composition of these blood-derived secretomes is a powerful tool for optimizing their angiogenic potency and, thus, clinical value as a wound healing therapy. Beyond controlling the incubation temperature and oxygen tension, which are key parameters during PBC conditioning, it may be possible that the proteomic profile is dependent on the nature of the nutritional medium used during this process. It is indeed known that optimal wound healing is dependent on nutritional status, as shown, for example, by a correlation between low serum albumin and the development of pressure ulcers [17]. There may also be direct effects of the supply of nutrients to the wound bed, as demonstrated, for example, by the ability of honey (which comprises a wide variety of active compounds, including flavonoids, phenolic acid, organic acids, enzymes, and vitamins) to improve the wound healing process [18]. Furthermore, some micronutrients, such as vitamins A, C, and E, may deactivate free radicals and potentially accelerate wound healing [19,20]. Studies have also shown that vitamin A functions as a hormone, altering the activity of epithelial cells, melanocytes, fibroblasts, and endothelial cells through its action on the family of retinoic acid receptors [21], while vitamin C promotes neutrophil and fibroblast activity and is required for optimal angiogenesis [19]. Proteins, on the other hand, are vital in keeping a positive nitrogen balance for all stages of the wound healing cascade, including fibroblast proliferation, collagen synthesis, angiogenesis, and immune response [20]. Beyond optimal cellular function, the tissue repair signaling itself is dependent on protein support, since growth factor production requires amino acid availability [22]. On the negative side of things, hyperglycemia correlates with stiffer blood vessels, which causes slower circulation and, consequently, reduced tissue oxygenation [23,24,25]. Indeed, chronic and acute hyperglycemia can trigger platelet activation [26,27], while in diabetic patients, the production of several growth factors involved in initiating and sustaining the healing process is compromised [25]; for example, vascular endothelial growth factor (VEGF) and transforming growth factor-beta (TGF-beta) protein expression is reduced in diabetic dermal wounds [28,29]. Recently, plasma lactate has emerged as an early indicator of aberrant metabolism, specifically, development of insulin resistance and diabetes mellitus [30]. In the context of wound healing, lactate accumulates as a consequence of both aerobic and anaerobic glycolysis following microcirculation disruption, immune activation, and increased cell proliferation [31]. Studies have repeatedly demonstrated its important contribution in tissue repair by promoting angiogenesis and collagen production [31,32,33,34].

Based on the established correlation between nutritional status and the wound healing response, we sought to examine whether PBC growth factor production, and consequently the angiogenic activity of hypoxia preconditioned secretomes, is also dependent on the type of nutritional medium used during PBC hypoxic conditioning. This was tested by substituting the autologous liquid components in HPP and HPS (i.e., plasma and serum, respectively) with various media (NaCl, phosphate buffered saline (PBS), Glucose 5% (G5%), and AIM V serum-free culture medium) in order to investigate their influence on the concentration of key pro- (VEGF-A, epidermal growth factor (EGF)) and anti-angiogenic protein factors (platelet factor-4 (PF-4), thrombospondin-1 (TSP-1)), before analyzing their ability to promote microvessel formation in vitro. Our findings suggest that it is indeed possible to influence the bioactivity of hypoxia preconditioned secretomes through medium substitution, potentially opening a new avenue for developing next-generation autologous growth factor cocktails for tissue repair and regeneration.

## 2. Results

### 2.1. Analysis of Lactate Concentration in Serum and HPS Depending on Exercise Level

**Figure 1:**
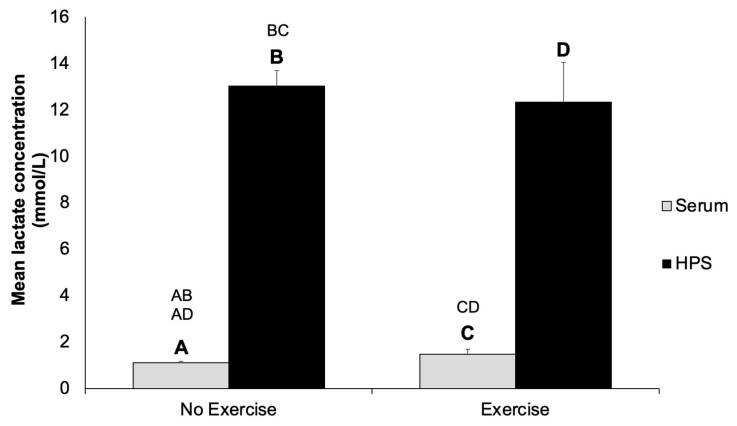
Quantitative analysis of lactate concentration in fresh serum and hypoxia preconditioned serum (HPS) in non-exercising and exercising subjects. Blood samples were obtained from subjects who exercised less than two hours per week (no-exercise group, n = 6) and from subjects who exercised more than two hours per week (exercise group, n = 6). Two-way ANOVA with Tukey’s multiple comparison test. Data points are means ± SD. Capital letter pairs over plots indicate statistical comparison of corresponding data points. For all pair comparisons, *p* < 0.0001.

We hypothesized that an increased lactate concentration would develop in hypoxia preconditioned serum (HPS), compared to baseline fresh serum, as a result of the exposure of PBCs to persistent hypoxia. Furthermore, we sought to identify a possible effect of regular exercise on the lactate concentration in both fresh serum and HPS. As expected, the lactate concentration increased significantly by approx. 12-fold in both non-exercising (1.10 ± 0.09 vs. 12.26 ± 0.84 mmol/L, *p* < 0.0001) and exercising (1.47 ± 0.23 vs. 12.33 ± 1.72 mmol/L, *p* < 0.0001) subjects over the 4-day incubation period (Figure 1), which indirectly confirmed the development of a hypoxic microenvironment during blood conditioning at 37 °C. Interestingly, there was no difference in lactate levels in either serum or HPS between the non-exercising and exercising group.

### 2.2. Analysis of Pro-Angiogenic Growth Factor Concentration (VEGF-A, EGF) in Various PBC Conditioning Media

To establish a growth factor concentration baseline, we first quantitatively analyzed via ELISA the concentration of angiogenesis-promoting growth factors (VEGF-A, EGF) in fresh plasma and serum and compared them to the hypoxia preconditioned plasma/serum (HPP/HPS) levels. We then analyzed the concentration of these protein factors in the different PBC conditioning media tested here as plasma/serum substitutes, i.e., hypoxia preconditioned normal saline (HPP/HPS-NaCl), hypoxia preconditioned phosphate buffered saline (HPP/HPS-PBS), hypoxia preconditioned Glucose 5% (HPP/HPS-G5%), and hypoxia preconditioned AIM V medium (HPP/HPS-AIM).

**Figure 2:**
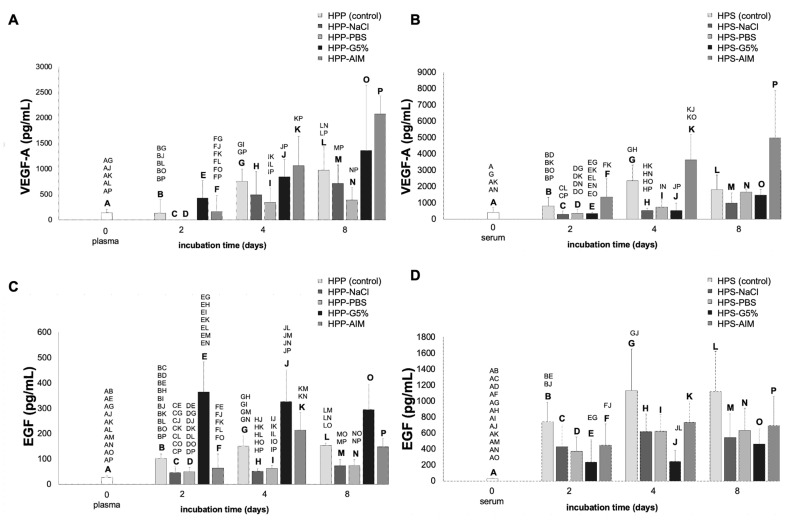
Quantitative analysis of pro-angiogenic growth factor (VEGF-A, EGF) concentration in various hypoxia preconditioned media. Plots showing the concentration of VEGF-A (pg/mL) (**A**,**B**) and EGF (pg/mL) (**C**,**D**) in fresh plasma and serum, as well as in hypoxia preconditioned plasma (HPP) and serum (HPS) and four different hypoxia preconditioned media substitutes: hypoxia preconditioned normal saline (HPP/HPS-NaCl), hypoxia preconditioned phosphate buffered saline (HPP/HPS-PBS), hypoxia preconditioned Glucose 5% (HPP/HPS-G5%), and hypoxia preconditioned AIM V medium (HPP/HPS-AIM), incubated for 2, 4, and 8 days. Blood donors: n = 4. Two-way repeated measures ANOVA with Tukey’s multiple comparison test. Data points are means ± SD. Capital letter pairs over plots indicate statistical comparison of corresponding data points. For all pair comparisons, *p* < 0.05.

The concentration of VEGF-A in HPP and HPS showed approx. a 5-fold increase after 4 days of incubation compared to the baseline level in fresh plasma (plasma vs. HPP: 142.63 vs. 757.00 pg/mL, *p* < 0.05) and serum (serum vs. HPS: 412.25 vs. 2350.33 pg/mL, *p* < 0.05) (Figure 2A,B). This similar relative increase implied that VEGF-A upregulation through PBC hypoxic conditioning was independent of platelet activation. Nonetheless, we found significantly higher levels of VEGF-A after conditioning without anticoagulants (4 day HPS vs. HPP: 2350.33 vs. 757.00 pg/mL, *p* < 0.05), while VEGF-A levels in fresh plasma and serum were not significantly different (142.63 vs. 412.25 pg/mL, *p* = 0.14), suggesting a moderate contribution from purely platelet-derived VEGF-A. Between day 4 and 8 of incubation, there was no further increase in the VEGF-A level in either HPP or HPS. 

In the next step of testing, we demonstrated an increase of the VEGF-A concentration from 2 to 4 and 8 days incubation in hypoxia preconditioned plasma substitutes (derived from anti-coagulated blood samples), with the highest level being achieved with HPP-AIM, which was twice as high compared to the HPP control on incubation day 8 (2079.25 vs. 974.50 pg/mL, *p* < 0.05) (Figure 2A). The observed increase in VEGF-A concentration, compared to baseline plasma levels, was least pronounced in HPP-PBS at all incubation days, with VEGF-A levels being significantly lower than the HPP control on incubation day 4 (348.5 vs. 757.00 pg/mL, *p* < 0.05) and day 8 (393.98 vs. 974.50 pg/mL, *p* < 0.05). A comparison of the VEGF-A concentration in hypoxia preconditioned serum substitutes (derived from coagulated blood samples) similarly indicated an increase from 2 to 4 and 8 days of incubation (Figure 2B). In contrast to all other media substitutes, the VEGF-A concentration of HPS-AIM samples exceeded the VEGF-A concentration of HPS control on each incubation day, although this difference was not statistically significant (*p* > 0.05). However, there were significant differences between HPS-AIM and other hypoxia preconditioned media VEGF-A levels, especially on incubation day 4, where a 7-fold increase was observed compared to HPS-NaCl (3637.00 vs. 517.00 pg/mL, *p* < 0.05) and HPS-G5% (3637.00 vs. 527.67 pg/mL, *p* < 0.05). This higher VEGF-A concentration in HPS-AIM persisted on incubation day 8, but was no longer significant. VEGF-A levels in all other hypoxia preconditioned serum substitutes (HPS-NaCl, HPS-PBS, and HPS-G5%) were below (day 2 and 4) or approx. at the same level (day 8) as the HPS control and hardly differed from one another (Figure 2B).

Similarly, the concentration of EGF in hypoxia preconditioned plasma (HPP) showed a 5-fold increase after 2 and 4 days of incubation compared to fresh plasma (101.90 vs. 27.50 pg/mL and 150.95 vs. 27.50 pg/mL, both *p* < 0.01), with no further significant difference between 4 and 8 days of incubation (Figure 2C). In contrast, a larger 40-fold increase in EGF concentration was observed in HPS compared to the baseline level in fresh serum (27.59 vs. 1133.13 pg/mL, *p* < 0.05) after 4 days of blood incubation. Importantly, the EGF concentration in HPS (range = 800–1200 pg/mL) was significantly higher than in HPP (range = 100–150 pg/mL) at all incubation time points, indicating the generation of a significant amount of platelet-derived EGF during conditioning. With regards to hypoxia preconditioned plasma substitutes, the EGF concentration of HPP-G5% exceeded that of the HPP control on all incubation days (day 2: 363.65 vs. 101.9 pg/mL; day 4: 327.10 vs. 150.95 pg/mL; day 8: 294.80 vs. 153.30 pg/mL, each *p* < 0.05). In contrast, the EGF concentration of HPP-NaCl and HPP-PBS was approx. half that of the HPP control on all incubation days (*p* < 0.05). HPP-AIM had a similar EGF level as the HPP control at all time points. A comparison of hypoxia preconditioned serum substitutes paradoxically showed that HPS-G5% had the lowest EGF concentration on all incubation days (Figure 2D), being significantly lower than the HPS control at 2 and 4 days of incubation (day 2: 235.63 vs. 742.5 pg/mL; day 4: 243.95 vs. 1133.13 pg/mL, both *p* < 0.05). HPS-NaCl, HPS-PBS, and HPS-AIM had a similar EGF concentration, which was consistently below that of the HPS control, albeit non-significantly (*p* > 0.05).

### 2.3. Analysis of Anti-Angiogenic Growth Factor Concentration (TSP-1, PF-4) in Various PBC Conditioning Media

**Figure 3:**
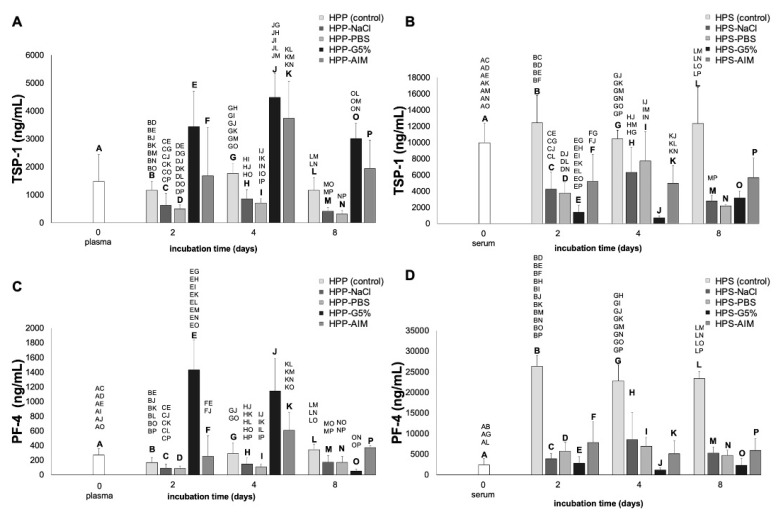
Quantitative analysis of anti-angiogenic growth factor (TSP-1, PF-4) concentration in various hypoxia preconditioned media. Plots showing the concentration of TSP-1 (ng/mL) (**A**,**B**) and PF-4 (ng/ml) (**C**,**D**) in fresh plasma and serum, as well as in hypoxia preconditioned plasma (HPP) and serum (HPS) and four different hypoxia preconditioned media substitutes: hypoxia preconditioned normal saline (HPP/HPS-NaCl), hypoxia preconditioned phosphate buffered saline (HPP/HPS-PBS), hypoxia preconditioned Glucose 5% (HPP/HPS-G5%), and hypoxia preconditioned AIM V medium (HPP/HPS-AIM), incubated for 2, 4, and 8 days. Blood donors: n = 4. Two-way repeated measures ANOVA with Tukey’s multiple comparison test. Data points are means ± SD. Capital letter pairs over plots indicate statistical comparison of corresponding data points. For all pair comparisons, *p* < 0.05.

As a next step, we sought to analyze the concentration of the platelet-derived angiogenic inhibitors TSP-1 and PF-4 in fresh plasma and serum and compared them to their hypoxia preconditioned counterparts (HPP/HPS), as well as to the different conditioning media substitutes previously tested. As shown in Figure 3A, the TSP-1 concentration of hypoxia preconditioned plasma (HPP) was comparable to that of fresh plasma and remained relatively stable over the entire incubation period of 8 days. This finding confirmed minimal platelet activation as a result of anticoagulated blood conditioning. In terms of the various hypoxia preconditioned plasma substitutes (HPP-NaCl, HPP-PBS, HPP-G5%, and HPP-AIM), the highest TSP-1 concentrations were achieved in HPP-G5% and HPP-AIM, with a peak level observed on incubation day 4 that was significantly higher than the HPP control (4490.00 vs. 1755.00 ng/mL, 3735.00 vs. 1755.00 ng/mL, both *p* < 0.05). HPP-NaCl and HPP-PBS showed the lowest TSP-1 concentration, with significant differences from the HPP control, on all incubation days (*p* < 0.05).

A comparison of the TSP-1 concentration in fresh serum and hypoxia preconditioned serum (HPS) of 2, 4, and 8 days incubation showed no significant differences (Figure 3B, *p* > 0.05), although levels here were approx. 10-fold higher than those achieved in plasma and HPP. Furthermore, the TSP-1 concentration in the HPS control was overall greater than in the various hypoxia preconditioned serum substitutes (HPS-NaCl, HPS-PBS, HPS-G5%, and HPS-AIM), and these differences were occasionally significant (*p* < 0.05). The lowest TSP-1 concentration at 2 and 4 days of incubation was seen in HPS-G5%, which starkly contrasted with the relatively high level seen in the anticoagulated state of HPP-G5%. Indeed, at these time points, the TSP-1 concentration in HPS-G5% was 5- to 10-fold lower than in the HPS control (*p* < 0.05) and 2- to 5-fold lower than in HPS-NaCl, HPS-PBS, and HPS-AIM (*p* < 0.05).

As expected from the TSP-1 data, the PF-4 concentration of plasma and HPP was comparable at all time points (Figure 3C), consistent with minimal platelet activation in these secretomes. In a similar pattern, the PF-4 concentration of HPP-G5% was approx. 5-fold higher than in the other anticoagulated blood-derived conditioning media (HPP-NaCl, HPP-PBS, and HPP-AIM) at 2 and 4 days of incubation (*p* < 0.05). Interestingly, this difference appeared to be reversed on incubation day 8, when the PF-4 concentration of HPP-G5% was significantly lower than in HPP-NaCl, HPP-PBS, and HPP-AIM (*p* < 0.05).

The approx. 10-fold higher PF-4 concentration observed in fresh serum (2397.00 ng/mL) compared to fresh plasma (270.10 ng/mL) confirmed the platelet activation as a result of blood coagulation (Figure 3C,D). An even larger increase (12-fold) in the PF-4 level was recorded with HPS following 2 days of blood incubation compared to fresh serum (26,376.75 vs. 2397.00 ng/mL, *p* < 0.05). This difference persisted on incubation days 4 and 8. The PF-4 concentration in the various coagulated blood-derived hypoxia preconditioned media (HP-NaCl, HP-PBS, HP-G5%, and HP-AIM) was comparable to fresh serum and generally significantly lower than in HPS at all time points (*p* < 0.05). Notably, and in agreement with the TSP-1 results, the lowest PF-4 concentration was seen in HPS-G5% (Figure 3D).

### 2.4. Effect of Various Conditioning Media on Microvessel Formation In Vitro

**Figure 4:**
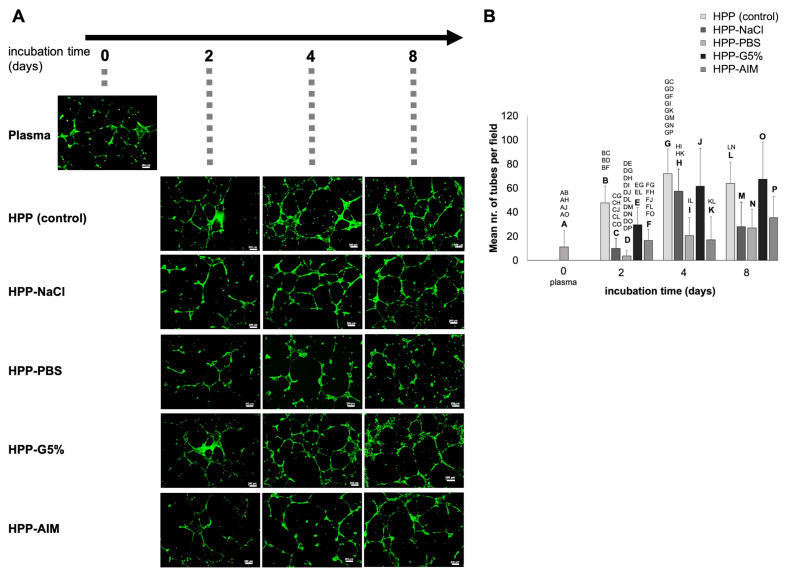
Effect of various anticoagulated blood-derived hypoxia preconditioned media on microvessel formation in endothelial cell cultures in vitro. (**A**) Panel showing representative images of the tube formation assay (12 h culture duration), carried out in the presence of anticoagulated blood-derived secretomes (fresh plasma; hypoxia preconditioned plasma (HPP)) and four different hypoxia preconditioned plasma substitutes: hypoxia preconditioned normal saline (HPP-NaCl), hypoxia preconditioned phosphate buffered saline (HPP-PBS), hypoxia preconditioned Glucose 5% (HPP-G5%), and hypoxia preconditioned AIM medium (HPP-AIM). Blood samples were obtained from 4 young, healthy subjects and conditioning was carried out over 2, 4, and 8 days prior to testing. Scale bars = 200 μm. (**B**) Plot showing the mean number of tubes formed in HUVEC cultures that were incubated for 12 h with anticoagulated blood-derived secretomes, as described in (**A**). Blood donors: n = 4. Two-way repeated measures ANOVA with Tukey’s multiple comparison test. Data points are means ± SD. Capital letter pairs over plots indicate statistical comparison of corresponding data points unless otherwise indicated. For all pair comparisons, *p* < 0.05.

Following an analysis of key pro- and anti-angiogenic protein factors, we moved on to investigate the ability of the blood-derived hypoxia preconditioned media to induce microvessel formation in human umbilical vein endothelial cell (HUVEC) in vitro cultures. With regards to anti-coagulated blood-derived secretomes, there was a 4-fold increase in the mean number of tubules observed in HPP cultures compared to fresh plasma, which was significant for 2-day HPP incubation (47.75 vs. 11.16, *p* < 0.001), while longer preconditioning periods (4 and 8 days) did not produce significant changes (*p* > 0.05) (Figure 4), indicating that an early plateau was reached in terms of the angiogenic response in this setting. At 2 days incubation, the tube formation results from HPP-G5% were comparable to the HPP control (*p* > 0.05), while HPP-NaCl, HPP-PBS, and HPP-AIM showed lower tube formation, with one-third to one-fourth the number of tubes observed in the HPP control and HPP-G5% cultures (*p* < 0.05). Nonetheless, even in these HPP-NaCl and HPP-PBS cultures, microvessel formation showed a 5-fold increase between 2 and 4 days of incubation (9.92 vs. 57.41; 3.67 vs. 20.50, both *p* < 0.01), which indicated that the duration of the conditioning period positively influenced the angiogenic activity of these secretomes to a somewhat greater extent than with the glucose or AIM medium substitution.

**Figure 5:**
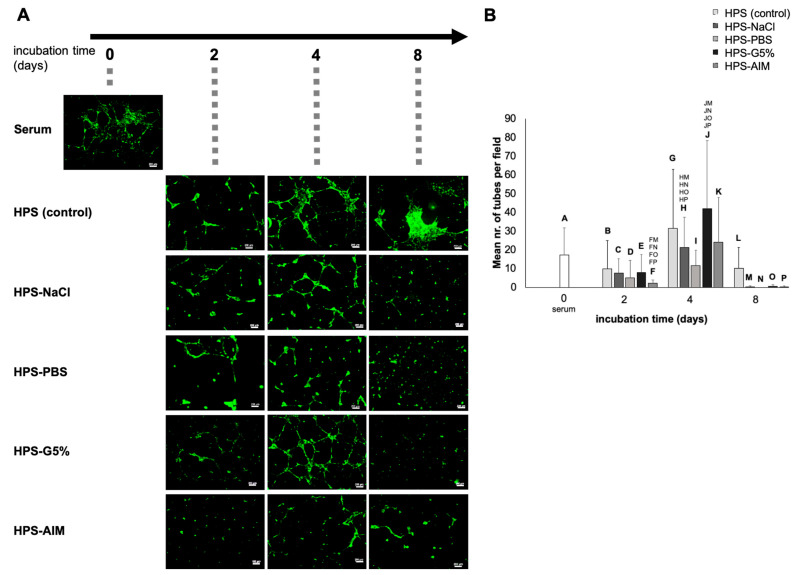
Effect of various coagulated blood-derived hypoxia preconditioned media on microvessel formation in endothelial cell cultures in vitro. (**A**) Panel showing representative images of the tube formation assay (12 h culture duration), carried out in the presence of coagulated blood-derived secretomes (fresh serum; hypoxia preconditioned serum (HPS)) and four different hypoxia preconditioned serum substitutes: normal saline (HPS-NaCl), hypoxia preconditioned phosphate buffered saline (HPS-PBS), hypoxia preconditioned Glucose 5% (HPS-G5%), and hypoxia preconditioned AIM medium (HPS-AIM). Blood samples were obtained from 4 young, healthy subjects and conditioning was carried out over 2, 4, and 8 days prior to testing. Scale bars = 200 μm. (**B**) Plot showing the mean number of tubes formed in HUVEC cultures that were incubated for 12 h with coagulated blood-derived secretomes, as described in (**A**). Blood donors: n = 4. Two-way repeated measures ANOVA with Tukey’s multiple comparison test. Data points are means ± SD. Capital letter pairs over plots indicate statistical comparison of corresponding data points unless otherwise indicated. For all pair comparisons, *p* < 0.05.

Despite the aforementioned differences in the protein quantification assays, there were no significant differences in terms of microvessel formation between the fresh serum- and HPS-incubated HUVEC cultures, regardless of the duration of the HPS preconditioning (Figure 5A,B). Furthermore, medium substitution with NaCl, PBS, G5%, or AIM did not appear to offer an advantage at 2 and 4 days of incubation when these secretomes were derived from coagulated blood. While the mean number of tubules generated by the 2- and 4-day preconditioned media (HPS-NaCl, HPS-PBS, HPS-G5%, and HPS-AIM) was comparable, there was a dramatic drop in the mean tube number observed in the cultures of 8-day preconditioned media, which was significant compared to the 4-day HPS-NaCl (all *p* < 0.05) and HPS-G5% (all *p* < 0.05) cultures. This suggested that, in contrast to the anticoagulated blood preconditioning, the length of the incubation period negatively affected the angiogenic activity of the coagulated blood-derived media.

## 3. Discussion

Hypoxia preconditioned blood-derived secretomes represent a new generation of autologous growth factor preparations that can be produced through extracorporeal conditioning of peripheral blood cells (PBCs) under wound-simulating conditions, namely, physiological temperature and hypoxia [5,9,10,11,12]. We had previously demonstrated that hypoxia preconditioned plasma (HPP) and serum (HPS) supply angiogenesis- and lymphangiogenesis-specific signaling, similar to that naturally produced within the wound microenvironment [5,6,7,12]. HPP and HPS organically differ with respect to their protein factor composition, since they correlate with distinct wound healing phases, the former having a direct correlation with the hypoxia-induced, angiogenesis-driven proliferative phase, while the latter also incorporates the platelet-derived hemostatic phase [6,10,12]. Despite their differences, the clinical utility of these secretomes harnesses their angiogenic activity, since they can both provide a useful tool for stimulating microvessel sprouting and lymphatic vessel formation on demand [5,6,7,8,12]. As such, they could play an important role in a modern therapeutic strategy that aims to improve local tissue perfusion and accelerate tissue healing where this is a delay or stagnation [9,10,12,13,14].

We hypothesized that medium substitution, which effectively gets rid of lactate already present in serum, as well as that which accumulates during incubation (through continuous substitution), may offer a means for optimizing the conditioning microenvironment for improved PBC function and growth factor production. Here, we showed a 12-fold increase in the lactate concentration of HPS compared to fresh serum as a result of blood conditioning (Figure 1). We also hypothesized that the PBCs of subjects who regularly exercise may produce less lactate as a result of cellular adaptation [35,36]. However, the lactate concentrations in both serum and HPS were comparable between the exercising and non-exercising groups. Hunt et al. had postulated that an increased concentration of lactate in wounds presented a major signal for collagen synthesis and wound repair [31,32]. Indeed, lactate actively participates in the healing process through the activation of several molecular pathways that collectively promote angiogenesis via endothelial cell migration [31,33,37,38,39] and tube formation in vitro [31,37,38,40], as well as the recruitment of circulating vascular progenitor cells in vivo [31,34,41]. These results indicate that the by-product “lactate”, which is inadvertently generated via the exposure of PBCs to hypoxia, may actually support the wound healing activity of these secretomes; however, further experiments with high versus low lactate conditions, while controlling for other angiogenic factors, are needed to verify its angiogenic effect in the various conditioning media.

The main thesis of this work is based on the notion that the net bioactivity of a growth factor-based regenerative therapy is effected through the balance of stimulatory and inhibitory signals. More specifically, it is known that a range of angiogenic inhibitors are of platelet origin and are thus released into coagulated blood-derived secretomes, e.g., HPS, as a result of platelet activation and degranulation [6,7,8,12,15]. In order to examine these effects, plasma and serum were substituted with different conditioning media, namely, hypoxia preconditioned normal saline (HPP/HPS-NaCl), phosphate buffered saline (HPP/HPS- PBS), Glucose 5% (HPP/HPS-G5%), and AIM V medium (HPP/HPS-AIM). These ‘novel’ compositions were subsequently analyzed in terms of the pro- and anti-angiogenic growth factor concentration and were tested for their ability to induce angiogenesis via tube formation assay. Quantitative analysis of pro-angiogenic growth factors (VEGF-A, EGF) in native hypoxia preconditioned secretomes (HPP/HPS) demonstrated a significant increase in VEGF-A concentration as a result of hypoxic conditioning, which became more evident after 4 days of incubation (Figure 2). However, none of the conditioning media substitutes tested appeared to offer a clear advantage in terms of this response, indicating that hypoxia regulation of VEGF-A expression may be the predominant factor determining its availability [42,43]. While there was a tendency for AIM V medium to induce more VEGF-A production, this effect only became significant after 8 days of incubation, in relation to the HPP control and other conditioning media, except for HPP-G5%. The glucose-containing medium HPP-G5% appeared to induce more platelet-derived VEGF-A and EGF secretion, as the increase in the concentration of these factors (especially EGF) was only detectable when platelet activation was kept at a minimum through blood anticoagulation (Figure 2A,C). EGF levels in HPP-G5% were significantly increased already after 2 days of incubation compared to the HPP control, while this level was comparable to the corresponding HPS-G5% EGF value (Figure 2C,D). In this regard, the VEGF-A and EGF levels in HPP-G5% and HPS-G5% were comparable at all incubation time points, indicating no further factor release through platelet activation as a result of clotting, while in the absence of glucose, the effect of clotting-induced platelet activation was apparent in the 3-fold increase in the VEGF-A level and the 5-fold increase in the EGF level in the 4-day incubated HPS control compared to the HPP control (Figure 2A,B). These findings highlight the catalytic role that glucose plays in platelet-mediated factor secretion, in agreement with the literature [44,45,46]. Clinically, there is also abundant evidence to support that hyperglycemia in diabetic patients is associated with increased plasma VEGF-A, which in turn may cause hypertension and several vascular complications in diabetic patients [47,48,49,50]. HPP/HPS-NaCl and HPP/HPS-PBS showed the lowest level of VEGF-A and EGF production for all incubation periods tested, regardless of platelet activation. This may be due to the lack of nutritional components that support adequate cell viability and protein synthesis. Another reason could be the absence of important electrolytes, such as magnesium and, more importantly, calcium, which are both known to promote platelet activation [51,52].

With regards to anti-angiogenic growth factors, our data showed that the TSP-1 and PF-4 levels in HPP were comparable to fresh plasma at all incubation time points, and were significantly lower than in HPS, indicating that hypoxic conditioning itself did not promote TSP-1/PF-4 expression, but rather platelet activation was the main source of these angiogenic inhibitors in these secretomes (Figure 3A,B). This is consistent with the higher TSP-1 and PF-4 levels observed in anticoagulated blood-derived HPP-G5%. The presence of glucose in the medium appeared to exert a strong stimulation of platelet activation, even in the background of previous heparin-mediated blood anticoagulation. However, similar to EGF, the levels of TSP-1 and PF-4 were significantly lower in HPS-G5% when compared to the HPS control at both 2 and 4 days of incubation (Figure 2 and Figure 3), suggesting that excess glucose interfered with platelet activation induced by clotting. This effect has been verified in the clinical setting, in which blood coagulation measurements via ROTEM (rotational thromboelastometry) are heavily interfered with if the blood sample is in a hyperglycemic state, resulting in an impaired clotting process, as evidenced by prolonged coagulation time measurements [53]. Altogether, these findings indicate that the elevated glucose concentration in the medium may significantly influence its final growth factor profile. Further investigation is required in order to decipher the relative contributions of glucose to PBC hypoxia-induced signaling and coagulation-mediated platelet factor secretion. 

The peak angiogenic response, analyzed here via in vitro tube formation assay, was observed with 4-day incubated secretomes and was generally stronger when these were derived from anti-coagulated rather than coagulated blood, likely due to the significantly higher concentration of angiogenic inhibitors in the latter (Figure 4 and Figure 5). Surprisingly, media substitution did not confer a significant improvement in terms of angiogenic response, compared to the HPP and HPS controls (Figure 4 and Figure 5). HPP/HPS-PBS consistently showed lower tube formation, in agreement with an overall weaker angiogenic growth factor response; however, even in these secretomes, there was a measurable increase in microvessels from 2 to 4 days, consistent with an increase in VEGF-A concentration (Figure 2A and Figure 4). Interestingly, HPP-G5% derived from anticoagulated blood induced a similar angiogenic response to the HPP control, despite the higher levels of the angiogenic inhibitors TSP-1 and PF-4, suggesting that this drawback was counterbalanced via an also higher concentration of pro-angiogenic factors, e.g., EGF, in HPP-G5%. This is consistent with our previous results, which demonstrated that the presence of type 1 and type 2 diabetes mellitus does not appear to significantly impact the angiogenicity of HPP and HPS in vitro (as compared to secretomes obtained from healthy subjects) [16]. These findings suggest that the application of hypoxia preconditioning may serve as a tool for improving the angiogenic potency of blood-derived secretomes, thus repairing the angiogenic dysfunction that is a direct consequence of the disease state of diabetes and hyperglycemia. It is also important to acknowledge, however, that an oversupply of VEGF-A (and other pro-angiogenic factors) or blood glucose can lead to vascular leakage by disruption of cell–cell adherence, as well as tight-gap junction molecular signaling [54,55]. Thus, the characterization of endothelial identity and the analysis of the functionality of induced vasculature (e.g., the presence of immature endothelial microvessels and increased vascular permeability), after stimulation through conditioning media, are key points for investigation that should be examined in future studies, employing, for example, immunohistochemical staining for vascular leakage markers (e.g., VE-Cadherin) or more complex experiments for detecting leakage of proteins/cells in an in vivo vascular permeability assay [56,57].

## 4. Materials and Methods

### 4.1. Ethical Approval

All blood donors provided written informed consent, as directed by the ethics committee of the Technical University Munich, Germany, which approved this study (File Nr.: 497/16S; amendment 2.0, date of approval: 18 September 2017). 

### 4.2. Analysis of Lactate Concentration during Hypoxic Preconditioning Depending on Fitness Level

Subjects were recruited in our clinic in 2021. We included 12 participants with an age of 34.5 ± 13.9 years (see demographics, Table 1). All participants were asked about their fitness level and number of exercising hours per week. Participants assigned to the “exercise group” were young healthy adults, without any medication and comorbidities, who exercised more than two hours per week. Participants who exercised less than two hours per week were assigned to the “no exercise” group. The age distribution was equal in both groups (*p* = 0.24). Smokers were defined as those who had smoked more than one cigarette in the past three months. Blood-derived secretomes from the participants were prepared as described in Section 2.3 and Section 2.4, and lactate levels were analyzed by blood gas analysis (Siemens Healthineers, Rapid Point 500, Erlangen, Germany).

### 4.3. Preparation of Blood Plasma/Serum and Hypoxia Preconditioned Plasma (HPP)/Serum (HPS) Samples

Peripheral venous blood (20 mL) was collected from young, healthy, non-smoking subjects (*n* = 4), who were not taking any medication and were without known comorbidities, in a 30 mL polypropylene syringe (Omnifix^®^, Braun AG, Melsungen, Germany) that contained no additive for normal serum and HPS preparation or was prefilled with 1 mL heparin (Medunasal^®^, Heparin 500 I.U. 5 mL ampoules, Sintetica^®^, Münster, Germany) for normal plasma and HPP preparation, under sterile and standardized conditions (Blood Collection Set 0.8 × 19 mm × 178 mm; Safety-Lok, CE 0050, BD Vacutainer, Franklin Lakes, New Jersey, USA). In the next step, the blood was centrifuged at 2000 rpm for 15 min at room temperature (22 °C). After centrifugation, the blood was separated into three layers, from bottom to top: red blood cell component (RBCs), buffy coat/clot, and plasma/serum, so that the top layer (plasma or serum) could be filtered into a new syringe. For HPP/HPS preparation, the protocol described by Hadjipanayi et al. was used [10,12]. Briefly, following blood sampling, a 0.2 µm pore filter was attached to the syringe (Sterifix^®^, CE 0123, Braun AG, Melsungen, Germany), and by pulling the plunger, 5 mL of air was drawn into the syringe through the filter. Subsequently, the filter was removed, and the capped syringe was placed upright in an incubator (37 °C/5% CO_2_) and incubated for 2, 4, or 8 days (blood incubation time) without prior centrifugation. Pericellular local hypoxia (~1% O_2_) was induced in situ through cell-mediated O_2_ consumption by controlling the blood volume per unit area (BVUA > 1 mL/cm^2^) and, consequently, the PBC seeding density in the syringe [10,15]. After the predefined incubation time, the blood was passively separated into three layers, from top to bottom: hypoxia preconditioned plasma (HPP)/hypoxia preconditioned serum (HPS), buffy coat/clot, and red blood cell (RBC) component, so that the top layer comprising HPP or HPS could be filtered (0.2 µm pore filter, Sterifix^®^, Braun AG, Melsungen, Germany) into a new syringe, removing cells/cellular debris. 

### 4.4. Preparation of Hypoxia Preconditioned Media Samples through Plasma and Serum Substitution

**Figure 6:**
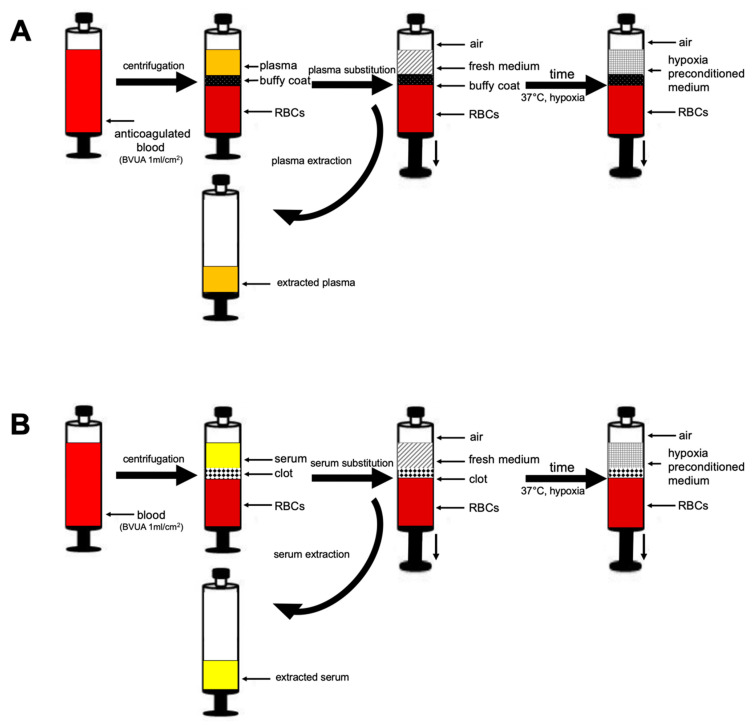
Preparation of hypoxia preconditioned samples through plasma and serum substitution. Preparation of fresh plasma (**A**) and serum (**B**). After centrifugation, the blood was separated into three layers, from bottom to top: red blood cell component (RBCs), buffy coat/clot, and plasma/serum, so that the top layer (plasma or serum) could be extracted into a new syringe. The same volume was then substituted in each case by saline (NaCl 0.9%), Phosphate buffered saline (PBS), Glucose 5% (G-5%), or AIM V serum-free medium (AIM V serum free), and 5 mL of air was drawn into the syringe through the filter and preconditioned under pericellular (local) hypoxia (~1% O_2_) and physiological temperature (37 °C) for 2 to 8 days. After the predefined incubation time, the growth factor-enriched hypoxia preconditioned medium (HPP/HPS-NaCl/-PBS/-G-5%/ -AIM) was separated by filtering into a new syringe, removing cells/cellular debris.

Plasma/serum were prepared as described above, from the same four subjects. After centrifugation, plasma/serum was removed (note: special care was taken not to disturb the buffy coat and clot in plasma and serum samples, respectively) under sterile conditions and the volume measured (Figure 6). The same volume was then substituted in each case by saline (NaCl 0.9%, B. Braun AG, Melsungen, Germany), Phosphate buffered saline (PBS) formulated without calcium and magnesium (Gibco, Thermo Fisher Science, Waltham, MA, USA), Glucose 5% solution containing glucose monohydrate (G-5%, B. Braun AG, Melsungen, Germany), or AIM V medium (AIM V serum-free medium containing L-glutamine, 50 µg/mL streptomycin sulfate, and 10 µg/mL gentamicin sulfate; Gibco, Thermo Fisher Science, Waltham, MA, USA). Then, a 0.2 µm pore filter was attached to the syringe (Sterifix^®^, CE 0123, Braun AG, Melsungen, Germany), and by pulling the plunger, 5 mL of air was drawn into the syringe through the 0.2 µm pore filter (Sterifix^®^, B Braun AG, Melsungen, Germany). Subsequently, the filter was removed, and the capped syringe was placed upright in an incubator (37 °C/5% CO_2_) and incubated for 2, 4, or 8 days (blood incubation time). Pericellular local hypoxia (~1% O_2_) was induced in situ through cell-mediated O_2_ consumption by controlling the blood volume per unit area (BVUA > 1 mL/cm^2^) and, consequently, the PBC seeding density in the syringe [10,15]. After the predefined incubation time, hypoxia preconditioned media (HPP/HPS-NaCl/-PBS/-G-5%/-AIM) were filtered (Sterifix^®^, Braun AG, Melsungen, Germany) into a new syringe, removing cells/cellular debris (Figure 6).

### 4.5. Quantitative Analysis of VEGF-A, EGF, TSP-1, PF-4 Concentration in Hypoxia Preconditioned Media

Blood-derived secretomes—fresh plasma and fresh serum, hypoxia preconditioned plasma (HPP) and serum (HPS), and hypoxia preconditioned media (HPP/HPS -NaCl/-PBS/-G-5%/-AIM) were sampled and analyzed by ELISA for vascular endothelial growth factor (VEGF-A), epidermal growth factor (EGF), thrombospondin-1 (TSP-1), and platelet-factor-4 (PF-4) (DY293B for VEGF-A, DY236 for EGF, DY3074 for TSP-1, DY795 for PF-4, Duoset, R&D Systems, Inc., Minneapolis, MN, USA), according to the manufacturer’s instructions. Factor concentrations in blood-derived secretomes/media were measured immediately after the predefined hypoxic incubation period (2, 4, or 8 days). All conditions were tested in triplicate per blood donor, and a total of four donors was taken for final evaluation.

### 4.6. Analysis of the Effect of Hypoxia Preconditioned Media on Microvessel Formation In Vitro

The angiogenic potential of blood-derived secretomes was tested in an in vitro angiogenesis assay by assessing their ability to induce microvessel formation in human umbilical vein endothelial cells (HUVECs, CellSystems, Troisdorf, Germany) seeded on factor-reduced Matrigel (BD, Heidelberg, Germany). HUVECs were seeded at a density of 10 × 10^3^/well, with 50 μL of test or control media added per well (μ-Slide Angiogenesis, Ibidi, Gräfelfing, Germany), and cultured in a 5% CO_2_/37 °C incubator for 12 h. Cells were then stained with Calcein AM (PromoKine, Heidelberg, Germany), and endothelial cell tube formation was observed with fluorescence and phase contrast microscopy. Assessment of the extent of capillary-like network formation was carried out by counting the number of tubes per field. Plasma and serum controls were tested immediately (day 0), while hypoxia preconditioned plasma/serum (HPP/HPS) and hypoxia preconditioned media (HPP/HPS -NaCl/-PBS/-G-5%/-AIM) were tested after the predefined incubation period (2, 4, or 8 days). All conditions were tested in triplicate per blood donor, and a total of four donors were taken for final evaluation.

### 4.7. Statistical Analysis

For statistical analysis, we used the GraphPad Prism 9 software. Data sets were analyzed by two-way analysis of variance (ANOVA), with subsequent comparisons using Tukey’s post hoc analysis. All values are expressed as means ± standard deviation (SD). A value of *p* < 0.05 was considered statistically significant.

## 5. Conclusions

Our data suggest that the presence of lactate in hypoxia preconditioned plasma and serum does not limit their bioactivity, which makes the need to remove it through medium substitution redundant. In addition to the availability of important electrolytes, e.g., calcium, the adjustment of blood glucose seems to have an influence on the pro- and anti-angiogenic growth factor profile of hypoxia preconditioned secretomes. Based on the results shown in this work, it appears that medium substitution of plasma/serum does not offer a clear benefit in terms of angiogenic potency, at least in vitro. Nonetheless, it may be possible to influence the secretomes’ pro-angiogenic factor (e.g., VEGF) levels via targeted medium substitution, especially with prolonged incubation periods. Medium substitution may, therefore, offer a useful tool for tailoring growth factor cocktails, based on peripheral blood hypoxic preconditioning, to specific applications.

## 6. Patents

Device-based methods for localized delivery of cell-free carriers with stress-induced cellular factors. (AU2013214187 (B2); 9 February 2017): Schilling Arndt, Hadjipanayi Ektoras, Machens Hans-Günther.

## Figures and Tables

**Table 1:** Demographic data: “No exercise” group included individuals with less than 2 h per week of exercise and “exercise” group included individuals with more than 2 h per week of exercise.

	No Exercise	Exercise
**Total number**	6	6
**Male/Female**	2/4	4/2
**Mean age ± SD** (years)	42.5 ± 24.5	29.8 ± 4.3
**Smoking** (Number of subjects)	1	0

## Data Availability

The data presented in this study are available on request from the corresponding authors.
